# Supplementary material for: The multi-subunit GID/CTLH E3 ubiquitin ligase promotes cell proliferation and targets the transcription factor Hbp1 for degradation
Source: eLife. 2018 Jun 18;7:e35528. doi: 10.7554/eLife.35528 (PMC6037477; doi:10.7554/eLife.35528)
Supplement: Figure 1—source data 1. [file elife-35528-fig1-data1.docx]

**Table 1–Source Data 1. List of WDR26-interactors identified by AP-MS. Related to Figure 1.**

| Protein ID | Protein | PSM | PSM Control | Unique Peptides | Coverage |
| --- | --- | --- | --- | --- | --- |
| Q9H7D7 | WD repeat-containing protein 26, WDR26 | 619 | 10 | 57 | 79.43 |
| Q96S59 | Ran-binding protein 9, RANBP9 | 68 | 0 | 23 | 45.68 |
| Q8IUR7 | Armadillo repeat-containing protein 8, ARMC8 | 71 | 0 | 15 | 62.85 |
| Q9H871 | Protein RMD5 homolog A, RMND5A | 45 | 0 | 26 | 79.28 |
| Q7L5Y9 | Macrophage erythroblast attacher, MAEA | 51 | 0 | 24 | 61.87 |
| Q9NWU2 | Glucose-induced degradation protein 8 homolog, GID8 | 23 | 0 | 13 | 70.18 |
| Q9UL63 | Muskelin OS=Homo sapiens, MKLN1 | 33 | 0 | 24 | 43.54 |
| Q8IVV7 | Glucose-induced degradation protein 4 homolog, GID4 | 20 | 0 | 13 | 63.00 |
| F5GY55 | DNA damage-binding protein 1, DDB1 | 9 | 12 | 12 | 10.53 |
| Q96G75 | Protein RMD5 homolog B, RMND5B | 8 | 0 | 6 | 22.90 |
| P62699 | Protein yippee-like 5 | 31 | 2 | 8 | 69.42 |
| A0A0D9SEU5 | Ran-binding protein 10, RANBP10 | 40 | 6 | 17 | 53.87 |
| H7C4M9 | Ubiquitin-conjugating enzyme E2 H | 1 | 0 | 1 | 24.19 |
