# Supplementary material for: The multi-subunit GID/CTLH E3 ubiquitin ligase promotes cell proliferation and targets the transcription factor Hbp1 for degradation
Source: eLife. 2018 Jun 18;7:e35528. doi: 10.7554/eLife.35528 (PMC6037477; doi:10.7554/eLife.35528)
Supplement: Figure 1—source data 2. [file elife-35528-fig1-data2.docx]

**Table 2–Source Data 1. List of Rmnd5a-interactors identified by AP-MS and SAINT analysis. Related to Figure 1.**

| PROTID | GENE | Rmnd5a_FC_A | Rmnd5a_SP | IP_Rmnd5a_1 | IP_Rmnd5a_2 | IP_Control_1 | IP_Control_2 |
| --- | --- | --- | --- | --- | --- | --- | --- |
| Q9UL63 | MKLN1 | 79.35 | 1 | 65 | 75 | 0 | 0 |
| Q8IVV7 | GID4 | 16.14 | 1 | 13 | 14 | 0 | 0 |
| G5E9V6 | ARMC8 | 37.37 | 1 | 30 | 35 | 0 | 0 |
| J3KNJ2 | EF | 6.03 | 1 | 4 | 5 | 0 | 0 |
| P55265 | ADAR | 7.8 | 1 | 7 | 5 | 0 | 0 |
| Q96S59 | RANBP9 | 35.73 | 1 | 60 | 70 | 1 | 0 |
| Q9H871 | RMND5A | 52.8 | 1 | 79 | 115 | 1 | 0 |
| Q9NWU2 | GID8 | 15.4 | 1 | 27 | 26 | 2 | 0 |
| Q9H7D7 | WDR26 | 26.25 | 1 | 87 | 108 | 0 | 10 |
| A0A087WV47 | AP-2 complex | 5.49 | 1 | 4 | 4 | 0 | 0 |
| O43464 | HTRA2 | 9.99 | 1 | 8 | 8 | 0 | 0 |
| Q8IUR7 | ARMC8 | 60.48 | 1 | 52 | 54 | 0 | 0 |
| Q7L5Y9 | MAEA | 66.27 | 1 | 60 | 56 | 0 | 0 |
| Q96E35 | ZMYND19 | 10.58 | 1 | 9 | 8 | 0 | 0 |
| A0A0D9SEU5 | RanBP10 | 39.62 | 1 | 32 | 37 | 0 | 0 |
| Q5T5P0 | SickleTail | 5.55 | 0.99 | 5 | 3 | 0 | 0 |
| P28838 | LAP3 | 6.14 | 0.99 | 6 | 3 | 0 | 0 |
| A3KMH1 | VWA8 | 4.9 | 0.99 | 3 | 4 | 0 | 0 |
| P62699 | YPEL5 | 4.62 | 0.99 | 9 | 11 | 2 | 2 |
| S4R341 | NOLC1 | 4.37 | 0.99 | 3 | 3 | 0 | 0 |
| K4DI93 | Cul4B | 4.31 | 0.96 | 2 | 4 | 0 | 0 |
| K7EIG7 | Myosin | 3.84 | 0.96 | 3 | 2 | 0 | 0 |
| H0YH88 | NAP1L1 | 3.84 | 0.96 | 3 | 2 | 0 | 0 |
| Q8N163 | CCAR2 | 3.25 | 0.93 | 2 | 2 | 0 | 0 |
| C9JG87 | MRPL39 | 3.25 | 0.93 | 2 | 2 | 0 | 0 |
| A0A0A0MRM8 | MYO6 | 3.31 | 0.93 | 3 | 3 | 0 | 1 |
| U3KQS7 | Hbp1 | 0.5 | 0.5 | 0 | 1 | 0 | 0 |
