# Supplementary material for: The multi-subunit GID/CTLH E3 ubiquitin ligase promotes cell proliferation and targets the transcription factor Hbp1 for degradation
Source: eLife. 2018 Jun 18;7:e35528. doi: 10.7554/eLife.35528 (PMC6037477; doi:10.7554/eLife.35528)
Supplement: Figure 1—source data 3. [file elife-35528-fig1-data3.docx]

**Table 3–Source Data 1. List of Armc8-interactors identified by AP-MS and SAINT analysis. Related to Figure 1.**

| PROTID | GENE | Armc_FC_A | Armc_SP | IP_Armc8_1 | IP_Armc8__2 | IP_Control_1 | IP_Control_2 |
| --- | --- | --- | --- | --- | --- | --- | --- |
| G5E9V6 | ARMC8 | 185.46 | 1 | 218 | 240 | 0 | 0 |
| C9JAA7 | ARMC8 | 97.18 | 1 | 118 | 121 | 0 | 0 |
| Q7L5Y9 | MAEA | 94.96 | 1 | 105 | 128 | 0 | 0 |
| R4GMX8 | RANBP10 | 71.78 | 1 | 89 | 87 | 0 | 0 |
| Q9UL63 | MKLN1 | 47.35 | 1 | 144 | 157 | 0 | 3 |
| Q8IVV7 | GID4 | 28.39 | 1 | 32 | 36 | 0 | 0 |
| K7EKE6 | LONP1 | 24.78 | 1 | 48 | 12 | 0 | 0 |
| Q96G75 | RMND5B | 22.29 | 1 | 28 | 25 | 0 | 0 |
| Q9NWU2 | GID8 | 22.28 | 1 | 39 | 43 | 0 | 1 |
| Q96S59 | RANBP9 | 20.99 | 1 | 173 | 166 | 5 | 6 |
| Q9H871 | RMND5A | 20.22 | 1 | 87 | 88 | 2 | 3 |
| Q8IUR7 | ARMC8 | 18.56 | 1 | 466 | 499 | 11 | 28 |
| Q9H7D7 | WDR26 | 17.45 | 1 | 165 | 162 | 5 | 8 |
| O43464 | HTRA2 | 16.31 | 1 | 18 | 20 | 0 | 0 |
| P62699 | YPEL5 | 15.44 | 1 | 20 | 16 | 0 | 0 |
| Q96E35 | ZMYND19 | 15.11 | 1 | 16 | 19 | 0 | 0 |
| O75306 | NDUFS2 | 11.38 | 1 | 17 | 9 | 0 | 0 |
| A0A024QZP7 | CDK1 | 11.13 | 1 | 9 | 16 | 0 | 0 |
| A3KMH1 | VWA8 | 10.46 | 0.98 | 22 | 2 | 0 | 0 |
| P16615 | ATP2A2 | 9.81 | 1 | 13 | 9 | 0 | 0 |
| P28838 | LAP3 | 9.61 | 1 | 21 | 12 | 1 | 0 |
| C9J625 | ARMC8 | 8.69 | 1 | 7 | 12 | 0 | 0 |
| Q9C0B9 | ZCCHC2 | 7.89 | 1 | 6 | 11 | 0 | 0 |
| A0A075B730 | EPPK1 | 7.79 | 1 | 11 | 6 | 0 | 0 |
| P62140 | PPP1CB | 7.48 | 1 | 6 | 10 | 0 | 0 |
| P51648 | ALDH3A2 | 7.44 | 1 | 8 | 8 | 0 | 0 |
| P82673 | MRPS35 | 7.31 | 1 | 19 | 15 | 1 | 1 |
| O75489 | NDUFS3 | 7.04 | 1 | 7 | 8 | 0 | 0 |
| Q9Y399 | MRPS2 | 7.02 | 1 | 8 | 7 | 0 | 0 |
| P61158 | ACTR3 | 7 | 1 | 9 | 6 | 0 | 0 |
| P82933 | MRPS9 | 6.59 | 0.99 | 17 | 5 | 1 | 0 |
| Q9BQ39 | DDX50 | 6.58 | 1 | 13 | 18 | 0 | 2 |
| O00629 | KPNA4 | 6.55 | 1 | 11 | 3 | 0 | 0 |
| P07195 | LDHB | 6.24 | 1 | 6 | 7 | 0 | 0 |
| Q9Y3D9 | MRPS23 | 6.2 | 1 | 8 | 5 | 0 | 0 |
| C9JN15 | PPIG | 6.2 | 1 | 8 | 5 | 0 | 0 |
| Q9NZL4 | HSPBP1 | 5.87 | 1 | 4 | 8 | 0 | 0 |
| Q15233 | NONO | 5.81 | 1 | 7 | 5 | 0 | 0 |
| Q6KB66 | KRT80 | 5.81 | 1 | 7 | 5 | 0 | 0 |
| F5GZS6 | SLC3A2 | 5.44 | 1 | 5 | 6 | 0 | 0 |
| P82664 | MRPS10 | 5.32 | 1 | 8 | 9 | 1 | 0 |
| Q9BZX2 | UCK2 | 4.65 | 1 | 3 | 6 | 0 | 0 |
| Q9UHQ7 | WBP5 | 4.65 | 1 | 3 | 6 | 0 | 0 |
| O60684 | KPNA6 | 4.61 | 1 | 5 | 4 | 0 | 0 |
| Q15392 | DHCR24 | 4.61 | 1 | 5 | 4 | 0 | 0 |
| Q7Z2W4 | ZC3HAV1 | 4.24 | 1 | 3 | 5 | 0 | 0 |
| P62937 | PPIA | 4.24 | 1 | 3 | 5 | 0 | 0 |
| Q99959 | PKP2 | 4.22 | 1 | 4 | 4 | 0 | 0 |
| Q8IUE6 | HIST2H2AB | 4.22 | 1 | 4 | 4 | 0 | 0 |
| Q9BTV4 | TMEM43 | 4.22 | 1 | 4 | 4 | 0 | 0 |
| O43592 | XPOT | 4.2 | 1 | 5 | 3 | 0 | 0 |
| Q96EY7 | PTCD3 | 4.19 | 1 | 15 | 9 | 1 | 2 |
| Q9H2V7 | SPNS1 | 4.18 | 0.98 | 6 | 2 | 0 | 0 |
| P13807 | GYS1 | 4.07 | 1 | 7 | 6 | 0 | 1 |
| P31153 | MAT2A | 3.94 | 0.98 | 6 | 11 | 1 | 1 |
| F8W7S5 | F8W7S5 | 3.85 | 0.98 | 2 | 5 | 0 | 0 |
| Q5QPL9 | RALY | 3.83 | 1 | 3 | 4 | 0 | 0 |
| O15173 | PGRMC2 | 3.83 | 1 | 3 | 4 | 0 | 0 |
| A0A087WUV8 | BSG | 3.83 | 1 | 3 | 4 | 0 | 0 |
| Q9P032 | NDUFAF4 | 3.83 | 1 | 3 | 4 | 0 | 0 |
| P24539 | ATP5F1 | 3.81 | 1 | 4 | 3 | 0 | 0 |
| S4R341 | S4R341 | 3.81 | 1 | 4 | 3 | 0 | 0 |
| P27824 | CANX | 3.81 | 1 | 4 | 3 | 0 | 0 |
| H3BPZ1 | H3BPZ1 | 3.67 | 0.99 | 6 | 5 | 1 | 0 |
| P12277 | CKB | 3.63 | 0.9 | 5 | 10 | 2 | 0 |
| Q07021 | C1QBP | 3.6 | 0.95 | 8 | 7 | 2 | 0 |
| P51398 | DAP3 | 3.56 | 1 | 17 | 16 | 3 | 3 |
| Q14684 | RRP1B | 3.12 | 0.97 | 5 | 4 | 1 | 0 |
| Q92665 | MRPS31 | 3.1 | 0.95 | 8 | 5 | 1 | 1 |
| F8VZY9 | F8VZY9 | 3.02 | 0.98 | 2 | 3 | 0 | 0 |
| O60725 | ICMT | 3.02 | 0.98 | 2 | 3 | 0 | 0 |
| Q9Y266 | NUDC | 3.02 | 0.98 | 2 | 3 | 0 | 0 |
| B7ZB02 | PRPS1 | 3.02 | 0.98 | 2 | 3 | 0 | 0 |
| B4DFG0 | DEK | 3.02 | 0.98 | 2 | 3 | 0 | 0 |
| B4DHE8 | MSI2 | 3 | 0.98 | 3 | 2 | 0 | 0 |
| Q9H0U6 | MRPL18 | 3 | 0.98 | 3 | 2 | 0 | 0 |
| Q07065 | CKAP4 | 2.88 | 0.95 | 16 | 10 | 4 | 2 |
| P43034 | PAFAH1B1 | 2.61 | 0.96 | 2 | 2 | 0 | 0 |
| P49411 | TUFM | 2.61 | 0.96 | 2 | 2 | 0 | 0 |
| Q9Y4W6 | AFG3L2 | 2.61 | 0.96 | 2 | 2 | 0 | 0 |
| E9PIA8 | PPT1 | 2.61 | 0.96 | 2 | 2 | 0 | 0 |
| Q9UJW0 | DCTN4 | 2.61 | 0.96 | 2 | 2 | 0 | 0 |
| D6RBE9 | ANXA5 | 2.61 | 0.96 | 2 | 2 | 0 | 0 |
| P46977 | STT3A | 2.61 | 0.96 | 2 | 2 | 0 | 0 |
| P51572 | BCAP31 | 2.61 | 0.96 | 2 | 2 | 0 | 0 |
| H7C1I0 | SLC35C1 | 2.61 | 0.96 | 2 | 2 | 0 | 0 |
| H0YNI7 | TLE-3 | 2.61 | 0.96 | 2 | 2 | 0 | 0 |
| P54709 | ATP1B3 | 2.61 | 0.96 | 2 | 2 | 0 | 0 |
| P32119 | PRDX2 | 2.61 | 0.96 | 2 | 2 | 0 | 0 |
| Q7Z3U7 | MON2 | 2.61 | 0.96 | 2 | 2 | 0 | 0 |
| Q96CP6 | GRAMD1A | 2.61 | 0.96 | 2 | 2 | 0 | 0 |
| O60381 | HBP1 | 2.18 | 0.5 | 3 | 0 | 0 | 0 |
| H7C4M9 | UBE2H | 1.41 | 0 | 0 | 1 | 0 | 0 |
