# Supplementary material for: The multi-subunit GID/CTLH E3 ubiquitin ligase promotes cell proliferation and targets the transcription factor Hbp1 for degradation
Source: eLife. 2018 Jun 18;7:e35528. doi: 10.7554/eLife.35528 (PMC6037477; doi:10.7554/eLife.35528)
Supplement: Figure 4—source data 1. [file elife-35528-fig4-data1.docx]

**Table 4–Source Data 1. List of Rmnd5a-interactors in the presence of MG132 identified by AP-MS and SAINT analysis. Related to Figure 4.**

| PROTID | GENE | Rmnd5A_FC_A | Rmnd5a_SP | IP_Rmnd5a_1 | IP_Rmnd5a_2 | IP_Control_1 | IP_Control_2 |
| --- | --- | --- | --- | --- | --- | --- | --- |
| Q9H871 | RMND5A | 128.04 | 1 | 197 | 196 | 0 | 0 |
| Q9H7D7 | WDR26 | 30.38 | 1 | 169 | 155 | 3 | 2 |
| P0DMV8 | HSPA1A | 2.19 | 1 | 144 | 151 | 46 | 39 |
| Q9UL63 | MKLN1 | 69.2 | 1 | 103 | 108 | 0 | 0 |
| Q96S59 | RANBP9 | 45.73 | 1 | 99 | 101 | 1 | 0 |
| Q7L5Y9 | MAEA | 43.03 | 1 | 67 | 63 | 0 | 0 |
| R4GMX8 | Ranbp10 | 33.97 | 1 | 51 | 51 | 0 | 0 |
| Q8IUR7 | ARMC8 | 30.42 | 1 | 46 | 45 | 0 | 0 |
| Q9NWU2 | GID8 | 23.31 | 1 | 36 | 33 | 0 | 0 |
| G5E9V6 | ARMC8 | 21.69 | 1 | 32 | 32 | 0 | 0 |
| A0A087X0X3 | HNRNPM | 18.78 | 1 | 28 | 27 | 0 | 0 |
| P54652 | HSPA2 | 18.78 | 1 | 28 | 27 | 0 | 0 |
| P49327 | FASN | 2.26 | 1 | 28 | 33 | 12 | 5 |
| Q99460 | PSMD1 | 2.96 | 1 | 27 | 31 | 13 | 0 |
| P58107 | Epiplakin | 19.42 | 1 | 25 | 32 | 0 | 0 |
| A0A024RCR6 | BAT3 | 9.86 | 1 | 22 | 32 | 2 | 0 |
| O95071 | UBR5 | 14.9 | 1 | 20 | 23 | 0 | 0 |
| Q16531 | DDB1 | 13.29 | 1 | 20 | 18 | 0 | 0 |
| P62195 | PSMC5 | 3.77 | 1 | 20 | 19 | 6 | 0 |
| O00232 | PSMD12 | 1.77 | 1 | 20 | 20 | 9 | 5 |
| P78527 | PRKDC | 7.44 | 1 | 19 | 21 | 2 | 0 |
| Q08380 | LGALS3BP | 6.93 | 1 | 18 | 19 | 2 | 0 |
| O14654 | IRS4 | 8.58 | 1 | 18 | 17 | 1 | 0 |
| P62699 | YPEL5 | 5.61 | 1 | 18 | 14 | 1 | 1 |
| P46821 | MAP1B | 3.36 | 1 | 16 | 11 | 3 | 1 |
| Q1KMD3 | HNRNPUL2 | 9.41 | 1 | 15 | 11 | 0 | 0 |
| Q7Z6Z7 | HUWE1 | 7.23 | 1 | 14 | 15 | 1 | 0 |
| Q7L5D6 | GET4 | 9.41 | 1 | 14 | 12 | 0 | 0 |
| P05787 | KRT8 | 11.34 | 1 | 14 | 18 | 0 | 0 |
| Q8IVV7 | GID4 | 7.79 | 1 | 13 | 8 | 0 | 0 |
| J3KTA4 | J3KTA4 | 1.82 | 1 | 13 | 15 | 5 | 4 |
| Q92598 | HSPH1 | 7.79 | 1 | 12 | 9 | 0 | 0 |
| P62244 | RPS15A | 9.08 | 1 | 11 | 14 | 0 | 0 |
| Q10567 | AP1B1 | 7.47 | 1 | 11 | 9 | 0 | 0 |
| I3L2C7 | GEMIN4 | 2.58 | 1 | 10 | 10 | 3 | 1 |
| Q9BUJ2 | HNRNPUL1 | 6.5 | 1 | 10 | 7 | 0 | 0 |
| P06493 | CDK1 | 8.75 | 1 | 10 | 14 | 0 | 0 |
| Q96E35 | ZMYND19 | 7.14 | 1 | 10 | 9 | 0 | 0 |
| C9J2Y9 | POLR2B | 7.46 | 1 | 9 | 11 | 0 | 0 |
| Q7Z406 | MYH14 | 6.17 | 1 | 9 | 7 | 0 | 0 |
| P61289 | PSME3 | 7.14 | 1 | 9 | 10 | 0 | 0 |
| Q7KZ85 | SUPT6H | 2.96 | 1 | 9 | 9 | 3 | 0 |
| H0YMZ1 | PSMA4 | 5.21 | 1 | 8 | 5 | 0 | 0 |
| P13807 | GYS1 | 2.73 | 1 | 8 | 6 | 1 | 1 |
| Q5T4S7 | UBR4 | 2.34 | 1 | 8 | 11 | 2 | 2 |
| E9PDE8 | HSPA4L | 5.53 | 1 | 7 | 7 | 0 | 0 |
| P49720 | PSMB3 | 5.53 | 1 | 7 | 7 | 0 | 0 |
| P33993 | MCM7 | 3.48 | 1 | 7 | 7 | 0 | 1 |
| Q99436 | PSMB7 | 2.25 | 1 | 7 | 7 | 2 | 1 |
| Q5STU3 | DDX39B | 4.56 | 1 | 6 | 5 | 0 | 0 |
| F5GWT4 | WNK1 | 4.56 | 1 | 6 | 5 | 0 | 0 |
| Q14CN4 | KRT72 | 4.88 | 1 | 6 | 6 | 0 | 0 |
| O95816 | BAG2 | 4.23 | 1 | 6 | 4 | 0 | 0 |
| Q13501 | SQSTM1 | 4.88 | 1 | 6 | 6 | 0 | 0 |
| P49721 | PSMB2 | 1.87 | 1 | 6 | 6 | 1 | 2 |
| G5E9A6 | USP11 | 3.59 | 1 | 5 | 3 | 0 | 0 |
| P12755 | SKI | 3.59 | 1 | 5 | 3 | 0 | 0 |
| O14744 | PRMT5 | 4.23 | 1 | 5 | 5 | 0 | 0 |
| P62899 | RPL31 | 3.91 | 1 | 5 | 4 | 0 | 0 |
| Q16769 | QPCT | 3.91 | 1 | 5 | 4 | 0 | 0 |
| P62750 | RPL23A | 3.91 | 1 | 5 | 4 | 0 | 0 |
| Q9NR09 | BIRC6 | 3.4 | 1 | 5 | 7 | 1 | 0 |
| B5MCW2 | RPL3 | 4.23 | 1 | 5 | 5 | 0 | 0 |
| P11441 | UBL4A | 3.91 | 1 | 5 | 4 | 0 | 0 |
| Q7Z7L1 | SLFN11 | 3.27 | 1 | 5 | 2 | 0 | 0 |
| P55735 | SEC13 | 3.27 | 1 | 5 | 2 | 0 | 0 |
| Q06323 | PSME1 | 2.46 | 1 | 5 | 4 | 0 | 1 |
| A5YKK6 | CNOT1 | 2.24 | 1 | 5 | 7 | 0 | 2 |
| Q99519 | NEU1 | 3.26 | 1 | 4 | 3 | 0 | 0 |
| Q96HS1 | PGAM5 | 3.26 | 1 | 4 | 3 | 0 | 0 |
| Q5T5P2 | KIAA1217 | 3.91 | 1 | 4 | 5 | 0 | 0 |
| G3V0E5 | TFRC | 4.23 | 1 | 4 | 6 | 0 | 0 |
| A2A3R7 | RPS6 | 3.59 | 1 | 4 | 4 | 0 | 0 |
| Q15021 | NCAPD2 | 4.23 | 1 | 4 | 6 | 0 | 0 |
| H7C0S3 | CDKN3 | 3.91 | 0.99 | 4 | 5 | 0 | 0 |
| O43765 | SGTA | 3.26 | 0.99 | 4 | 3 | 0 | 0 |
| Q86U86 | PBRM1 | 3.91 | 0.99 | 4 | 5 | 0 | 0 |
| Q3ZCQ8 | TIMM50 | 3.59 | 0.99 | 4 | 4 | 0 | 0 |
| Q9UL15 | BAG5 | 2.94 | 0.99 | 4 | 2 | 0 | 0 |
| O60341 | KDM1A | 2.94 | 0.99 | 4 | 2 | 0 | 0 |
| E7EPN9 | PRRC2C | 3.58 | 0.99 | 3 | 5 | 0 | 0 |
| B8QGS9 | PKP2 | 2.94 | 0.99 | 3 | 3 | 0 | 0 |
| Q9UNE7 | STUB1 | 2.94 | 0.99 | 3 | 3 | 0 | 0 |
| H3BUH7 | ALDOA | 2.94 | 0.99 | 3 | 3 | 0 | 0 |
| O14497 | ARID1A | 2.94 | 0.99 | 3 | 3 | 0 | 0 |
| H7C4S2 | Hbp1 | 3.26 | 0.99 | 3 | 4 | 0 | 0 |
