# Supplementary material for: The multi-subunit GID/CTLH E3 ubiquitin ligase promotes cell proliferation and targets the transcription factor Hbp1 for degradation
Source: eLife. 2018 Jun 18;7:e35528. doi: 10.7554/eLife.35528 (PMC6037477; doi:10.7554/eLife.35528)
Supplement: Figure 4—source data 2. [file elife-35528-fig4-data2.docx]

**Table 5–Source Data 1. List of Hbp1-interactors identified by AP-MS and SAINT analysis. Related to Figure 4.**

| PROTID | GENE | HBP1_FC_A | HBP1_SP | IP_HBP1_1 | IP_HBP1_2 | IP_ControlHEK_1 | IP_ControlHEK_2 |
| --- | --- | --- | --- | --- | --- | --- | --- |
| Q9H0E3 | SAP130 | 11.92 | 1 | 18 | 17 | 0 | 0 |
| Q9NUD5 | ZCCHC3 | 5.45 | 1 | 9 | 6 | 0 | 0 |
| P18077 | RPL35A | 3.97 | 1 | 6 | 4 | 0 | 0 |
| O76021 | RSL1D1 | 7.52 | 1 | 11 | 10 | 0 | 0 |
| Q9H6R4 | NOL6 | 6.48 | 1 | 10 | 8 | 0 | 0 |
| Q13823 | GNL2 | 6.03 | 1 | 8 | 8 | 0 | 0 |
| Q9UL63 | MKLN1 | 25.55 | 1 | 48 | 34 | 0 | 0 |
| O00267 | SUPT5H | 2.71 | 1 | 13 | 16 | 5 | 2 |
| Q8WWQ0 | PHIP | 8.1 | 1 | 10 | 12 | 0 | 0 |
| Q9NRX1 | PNO1 | 4.89 | 1 | 8 | 7 | 1 | 0 |
| Q9BZE4 | GTPBP4 | 8.98 | 1 | 27 | 22 | 4 | 0 |
| Q92615 | LARP4B | 3.34 | 1 | 5 | 3 | 0 | 0 |
| Q99848 | EBNA1BP2 | 5.73 | 1 | 12 | 10 | 0 | 0 |
| Q9H160 | ING2 | 3.74 | 1 | 5 | 4 | 0 | 0 |
| Q5JTH9 | RRP12 | 7.14 | 1 | 20 | 12 | 3 | 0 |
| O60832 | DKC1 | 4.69 | 1 | 11 | 3 | 0 | 0 |
| Q13610 | PWP1 | 5.86 | 1 | 9 | 7 | 0 | 0 |
| Q12899 | TRIM26 | 5.58 | 1 | 6 | 8 | 0 | 0 |
| O60381 | HBP1 | 36.15 | 1 | 52 | 58 | 0 | 0 |
| A2A2V2 | RBM34 | 3.79 | 1 | 7 | 3 | 0 | 0 |
| C9JQV0 | C7orf50 | 2.99 | 1 | 4 | 4 | 0 | 0 |
| Q9ULX3 | NOB1 | 3.4 | 1 | 7 | 7 | 0 | 0 |
| E7EQZ4 | SMN1;SMN2 | 3.34 | 1 | 5 | 3 | 0 | 0 |
| E9PJF5 | BRMS1 | 3.97 | 1 | 6 | 4 | 0 | 0 |
| Q9NP50 | FAM60A | 5.63 | 1 | 8 | 7 | 0 | 0 |
| Q02880 | TOP2B | 7.45 | 1 | 22 | 12 | 3 | 0 |
| Q49A26 | GLYR1 | 6.08 | 1 | 10 | 7 | 0 | 0 |
| Q9NXF1 | TEX10 | 4.59 | 1 | 20 | 13 | 5 | 0 |
| Q8IY81 | FTSJ3 | 6.26 | 1 | 15 | 7 | 1 | 0 |
| B1ANR0 | PABPC4 | 6.85 | 1 | 15 | 12 | 0 | 0 |
| I3L1L3 | I3L1L3 | 8.55 | 1 | 33 | 13 | 4 | 0 |
| Q96S59 | RANBP9 | 11.66 | 1 | 39 | 26 | 1 | 0 |
| Q5PSV4 | BRMS1L | 9.63 | 1 | 15 | 13 | 0 | 0 |
| K4DI93 | K4DI93 | 5.81 | 1 | 7 | 8 | 0 | 0 |
| P11388 | TOP2A | 8.32 | 1 | 38 | 27 | 5 | 0 |
| Q9HAJ7 | SAP30L | 4.15 | 1 | 5 | 5 | 0 | 0 |
| Q8WXF0 | SRSF12 | 3.74 | 1 | 5 | 4 | 0 | 0 |
| Q15477 | SKIV2L | 6.44 | 1 | 8 | 9 | 0 | 0 |
| Q9NQS7 | INCENP | 4.6 | 1 | 7 | 5 | 0 | 0 |
| Q9UIG0 | BAZ1B | 5.7 | 1 | 31 | 30 | 6 | 0 |
| Q9H7L9 | SUDS3 | 9.05 | 1 | 16 | 11 | 0 | 0 |
| A0A0D9SEM4 | SRSF4 | 5.13 | 1 | 4 | 8 | 0 | 0 |
| Q9UII4 | HERC5 | 6.31 | 1 | 11 | 7 | 0 | 0 |
| Q8TDN6 | BRIX1 | 6.97 | 1 | 18 | 14 | 1 | 0 |
| P55201 | BRPF1 | 4.37 | 1 | 6 | 5 | 0 | 0 |
| P29374 | ARID4A | 10.84 | 1 | 15 | 16 | 0 | 0 |
| Q9UKD2 | MRTO4 | 6.8 | 1 | 6 | 11 | 0 | 0 |
| Q71RC2 | LARP4 | 3.34 | 1 | 5 | 3 | 0 | 0 |
| Q9H871 | RMND5A | 7.02 | 1 | 20 | 14 | 0 | 0 |
| Q5SSJ5 | HP1BP3 | 8.37 | 1 | 13 | 11 | 0 | 0 |
| Q6P158 | DHX57 | 14.44 | 1 | 22 | 21 | 0 | 0 |
| Q4LE39 | ARID4B | 18.62 | 1 | 28 | 28 | 0 | 0 |
| Q16769 | QPCT | 5.58 | 1 | 6 | 8 | 0 | 0 |
| Q9NWU2 | GID8 | 4.28 | 1 | 9 | 9 | 0 | 0 |
| Q07020 | RPL18 | 5.63 | 1 | 8 | 7 | 0 | 0 |
| B7Z6D5 | DDX27 | 3.79 | 1 | 7 | 3 | 0 | 0 |
| O15226 | NKRF | 2.51 | 1 | 3 | 3 | 1 | 0 |
| F5H018 | RAN | 3.52 | 1 | 4 | 4 | 0 | 0 |
| Q9NWT1 | PAK1IP1 | 4.37 | 1 | 6 | 5 | 0 | 0 |
| Q13619 | CUL4A | 7.47 | 1 | 9 | 11 | 0 | 0 |
| Q96ST3 | SIN3A | 33.98 | 1 | 99 | 77 | 4 | 0 |
| P46777 | RPL5 | 2.04 | 1 | 39 | 40 | 14 | 5 |
| P46779 | RPL28 | 4.15 | 1 | 5 | 5 | 0 | 0 |
| P62753 | RPS6 | 2.74 | 1 | 14 | 15 | 0 | 3 |
| P46821 | MAP1B | 7.52 | 1 | 23 | 10 | 1 | 0 |
| Q9BVS4 | RIOK2 | 2.89 | 1 | 3 | 3 | 0 | 0 |
| O60506 | SYNCRIP | 3.56 | 1 | 6 | 3 | 0 | 0 |
| P23258 | TUBG1 | 4.24 | 1 | 9 | 3 | 0 | 0 |
| J3KT86 | J3KT86 | 2.72 | 1 | 3 | 4 | 0 | 0 |
| Q8IUR7 | ARMC8 | 11.39 | 1 | 21 | 14 | 0 | 0 |
| Q9H6W3 | Q9H6W3 | 3.11 | 1 | 4 | 3 | 0 | 0 |
| A0A087WV66 | A0A087WV66 | 25.11 | 1 | 61 | 35 | 1 | 0 |
| O75446 | SAP30 | 3.8 | 1 | 6 | 5 | 1 | 0 |
| A0A0D9SEU5 | RANBP10 | 8.46 | 1 | 17 | 9 | 0 | 0 |
| P63173 | RPL38 | 7.11 | 1 | 11 | 9 | 0 | 0 |
| P18583 | SON | 4.16 | 0.99 | 20 | 12 | 5 | 0 |
| O75367 | H2AFY | 2.61 | 0.99 | 14 | 11 | 0 | 4 |
| Q5SY16 | NOL9 | 3.43 | 0.99 | 11 | 7 | 3 | 0 |
| D3YTB1 | RPL32 | 2.19 | 0.99 | 9 | 9 | 3 | 1 |
| E7EW05 | SDAD1 | 2.93 | 0.99 | 5 | 2 | 0 | 0 |
| Q13547 | HDAC1 | 3.52 | 0.99 | 29 | 20 | 9 | 0 |
| Q9Y3A4 | RRP7A | 2.71 | 0.99 | 4 | 2 | 0 | 0 |
| Q15397 | KIAA0020 | 4.06 | 0.99 | 10 | 2 | 0 | 0 |
| Q16576 | RBBP7 | 6.18 | 0.99 | 26 | 19 | 9 | 0 |
| Q92900 | UPF1 | 2.94 | 0.99 | 6 | 4 | 0 | 0 |
| Q9H4L4 | SENP3 | 3.35 | 0.99 | 9 | 4 | 0 | 0 |
| O14646 | CHD1 | 4.28 | 0.99 | 17 | 11 | 5 | 0 |
| Q8TDD1 | DDX54 | 3.16 | 0.99 | 6 | 2 | 0 | 0 |
| A8MV53 | PPAN | 2.48 | 0.99 | 3 | 2 | 0 | 0 |
| Q9Y2X3 | NOP58 | 3.83 | 0.99 | 9 | 2 | 0 | 0 |
| Q6PKG0 | LARP1 | 3.92 | 0.99 | 32 | 16 | 3 | 0 |
| P36578 | RPL4 | 2.52 | 0.99 | 27 | 28 | 10 | 0 |
| H7BXY3 | H7BXY3 | 4.24 | 0.98 | 31 | 17 | 7 | 0 |
| H3BR04 | H3BR04 | 2.26 | 0.98 | 2 | 2 | 0 | 0 |
| Q8NC51 | SERBP1 | 2.95 | 0.98 | 21 | 13 | 4 | 0 |
| Q2TB10 | ZNF800 | 2.26 | 0.98 | 2 | 2 | 0 | 0 |
| Q9H7D7 | WDR26 | 5.3 | 0.98 | 32 | 16 | 0 | 0 |
| Q8IVV7 | GID4 | 1.9 | 0.5 | 4 | 0 | 0 | 0 |
