# Supplementary material for: The multi-subunit GID/CTLH E3 ubiquitin ligase promotes cell proliferation and targets the transcription factor Hbp1 for degradation
Source: eLife. 2018 Jun 18;7:e35528. doi: 10.7554/eLife.35528 (PMC6037477; doi:10.7554/eLife.35528)
Supplement: Supplementary file 1 [file elife-35528-supp1.docx]

# Supplementary Material and Methods:

**List of plasmids used in this study. Related to Material and Methods.**

| Plasmid NAME | Insert | Backbone |
| --- | --- | --- |
| pFL179 | HSS-HBP1 | pcDNA5-FRT |
| pFL194 | GST-Tev-Ube2E2 | pGEX-Tev |
| pFL195 | GST-Tev-Ube2E1 | pGEX-Tev |
| pFL203 | WDR26 | pFL MultiBac |
| pFL204 | AviTAG-FLAG-Hbp1 | pFL MultiBac |
| pFL206 | GID8/Twa1 | pFL MultiBac |
| pFL207 | WDR26 FLAG-GID9 | pFL MultiBac |
| pFL208 | Rmnd5a | pFL MultiBac |
| pFL210 | Twa1 and MKLN1 | pFL MultiBac |
| pFL212 | GST-Tev-Ube2H | pGEX-Tev |
| pFL213 | GST-Tev-UbcH7 | pGEX-Tev |
| pFL215 | MKLN1 | pFL MultiBac |
| pFL216 | Armc8 | pFL MultiBac |
| pFL219 | Rmnd5a RanBP9 | pFL MultiBac |
| pFL220 | GID4 and GID5 | pFL MultiBac |
| pFL222 | Ube2G2 | pGEX-Tev |
| pFL227 | 2xSTREP-Precission-Armc8 | pcDNA5-FRT |
| pFL228 | pFL-PSPARMC8 | pFL MultiBac |
| pFL229 | yFbp1-Myc | pcDNA-3.1 |
| pFL230 | GID4 | pFL MultiBac |
| pFL234 | Hbp1-FLAG | pFL MultiBac |
| pFL235 | GID4 Armc8 | pFL MultiBac |
| pFL236 | GID4 -2xSTREP-PrecissionArmc8 | pFL MultiBac |
| pFL237 | HSS-Armc8 | pcDNA5-FRT |
| pFL238 | HSS-Rmnd5a | pcDNA5-FRT |
| pDD18 | HSS-WDR26-isoform 2 | pTRIPZ |
| pDD90 | WDR26 resistant to si_DD30/si_DD34 isoform 2 | pTRIPZ |
| pDD149 | Hbp1 canonical (isoform 2) | pTRIPZ |
| pDD126 | Hbp1 Δ1-10 | pTRIPZ |
| pDD127 | P11/G Hbp1 Δ1-10 | pTRIPZ |
| pDD128 | K16/R Hbp1 Δ1-10 | pTRIPZ |
| pDD100 | sgDD1 | lentiCRISPR v2 |
| pDD101 | sgDD2 | lentiCRISPR v2 |
| pDD102 | sgDD3 | lentiCRISPR v2 |
| pDD121 | sgDD4 | lentiCRISPR v2 |
| pDD122 | sgDD5 | lentiCRISPR v2 |
| pDD123 | sgDD6 | lentiCRISPR v2 |
| pDD124 | sgDD7 | lentiCRISPR v2 |
| pDD125 | sgDD8 | lentiCRISPR v2 |
| pDD116 | sgDD9 | lentiCRISPR v2 |
| pDD105 | sgDD10 | lentiCRISPR v2 |
| pDD114 | HSS-MAEA | pTRIPZ |
| pDD129 | Twa1-HSS | pTRIPZ |

**List of siRNAs used in this study. Related to Material and Methods.**

| siRNA name | Gene | Sequence |
| --- | --- | --- |
| HUMAN |  |  |
| si_Control (ASN) |  |  |
| si_DD33 | WDR26 | GCC ACA AGA UAC UAA CAA A |
| si_DD34 | WDR26 | GAACAUAGUACAAGAAGAU |
| si_DD30 | WDR26 | CUACCAAAUUCCGAAAUCA |
| si_DD43 | WDR26 | GCAAGUUGAUCCGGAUACA |
| si_DD38 | MAEA | GACGUGAUGAACGAGAACA |
| si_DD40 | MAEA | GUCAAGAUGAUAAAGUCGU |
| si_DD41 | Twa1 | CCGAUGAAAUCACGAAAGA |
| si_DD42 | Twa1 | CUUGGACACAAACCGGUAU |
| si_DD31 | Rmnd5a | CACCAUAUGUUCACCUACU |
| si_DD36 | Hbp1 | GUGUCCUCUUCUUCGAAGA |
| si_DD37 | Hbp1 | GAAUUGGCAAAUAUCGCGA |
| si_DD32 | Hbp1 | ACUGUGAGUGCCACUUCUC |
|  |  |  |
| MOUSE |  |  |
| M-046913-01-0005 | mGID8 | purchased from Dharmacon |
| M-048646-01-0005 | mMAEA | purchased from Dharmacon |
| M-050001-01-0005 | mRanBP9 | purchased from Dharmacon |
| M-046378-00-0005 | mRmnd5a | purchased from Dharmacon |
| M042620-00-0005 | mGID4 | purchased from Dharmacon |
| M-047124-01-0005 | mArmc8 | purchased from Dharmacon |
| M053449-01-0005 | mWDR26 | purchased from Dharmacon |

**List of qRT-PCR primers used in this study. Related to Material and Methods.**

| Oligo name | Gene | Sequence |
| --- | --- | --- |
| HUMAN |  |  |
| oDD133 | SDHA | tccactacatgacggagcag |
| oDD134 | SDHA | ccatcttcagttctgctaaacg |
| oDD150 | Hbp1 | taatggcgacgggtttgt |
| oDD151 | Hbp1 | ttcattcctgaggctctcttg |
| oDD121 | Cyclin D1 | gctgtgcatctacaccgaca |
| oDD122 | Cyclin D1 | ttgagcttgttcaccaggag |
|  |  |  |
| MOUSE |  |  |
| Forward | mGID8 | TGA TGC AGA GGC AAA AGG TA |
| Reverse | mGID8 | CTT GGG TGT GGA CTC TCG AT |
| Forward | mMAEA | ATT GAA CTT GTC CGG CAG A |
| Reverse | mMAEA | TGA AGT GCT TTC TTG CAT GTC |
| Forward | mRanBP9 | AGC TAC AAG CCA TGA GTG AGC |
| Reverse | mRanBP9 | GCT AGT AAG CTG AAT GCG TCC |
| Forward | mRmnd5a | GCC TCA AGC ACG AGA TCC |
| Reverse | mRmnd5a | GAA AGT GTC CCT GAT AAT TCA |
| Forward | mGID4 | CTG AAA AAT GGG GAT TAT GTTC |
| Reverse | mGID4 | GTG GTC CGG GAC TAG GAA C |
| Forward | mArmc8 | CTT GAA TTT TCT CCA AGT AAA |
| Reverse | mArmc8 | CTG TGT CAA CCC ACA GAG C |
| Forward | mWDR26 | TCC GAT GAG GAT GTC ATT AGG |
| Reverse | mWDR26 | TGC ATG AGG AGA TCA ACA GTC |

**List of guide RNAs used in this study. Related to Material and Methods.**

| Guide name | Gene | Sequence |
| --- | --- | --- |
| gDD1 | WDR26 | GGCTGTGGCCGCTACTTCGG |
| gDD2 | Wdr26 | CAATAACGGGGTTCCCGGCG |
| gDD3 | Wdr26 | AAGTATCCCTTACCGCCAGC |
| gDD4 | MAEA | GCGGCCAAAACATTAGCGGG |
| gDD5 | MAEA | TCATTCTAAGAAGTGCGACG |
| gDD6 | MAEA | GCTGACCACGGAGTCCACGG |
| gDD7 | MAEA | CGTTTGTTCAGCGTCTCGTA |
| gDD8 | MAEA | GGAGTGGCTCTCCGTTCAGC |
| gDD9 | Luciferase | CCCGAATCTCTATCGTGCGG |
| gDD10 | MAEA | GGCCAAAACATTAGCGGGCG |

**List of yeast strains used in this study. Related to Material and Methods**

| Strain name | Gene | Background S288C |
| --- | --- | --- |
| FLY66 | Fbp1-TAP | MATa; leu2, ura3, his3, lys2 |
| FLY67 | *∆moh1*, Fbp1-TAP | Mata; leu2, ura3, Fbp1-TAP::HIS3, *∆moh1*::KanMX |
| FLY68 | *∆gid4*, Fbp1-TAP | Mata; leu2, ura3, Fbp1-TAP::HIS3, *∆gid4*::KanMX |

**List of cell lines used in this study. Related to Material and Methods.**

| Plasmid NAME | Insert | Backbone | Cell type |
| --- | --- | --- | --- |
| cFL179 | HSS-HBP1 | pcDNA5-FRT | HEK 293 |
| cFL237 | HSS-Armc8 | pcDNA5-FRT | HEK 293 |
| cFLL238 | HSS-Rmnd5a | pcDNA5-FRT | HEK 293 |
| cDD12 | MAEA KO | lentiCRISPR v2 | HEK 293 |
| cDD13 | WDR26 KO | lentiCRISPR v2 | Hela Kyoto |
| cDD24 | pDD116/pDD105 (control sgRNAs) | lentiCRISPR v2 | RPE-adapted |
| cDD25 | pDD100/pDD101 (WDR26 sgRNAs) | lentiCRISPR v2 | RPE-adapted |
| cDD26 | pDD123/pDD125 (MAEA sgRNAs) | lentiCRISPR v2 | RPE-adapted |
| cDD149 | Hbp1 canonical | pTRIPZ | Hela Kyoto |
| cDD126 | Hbp1 Δ1-10 | pTRIPZ | Hela Kyoto |
| cDD127 | P11/G Hbp1 Δ1-10 | pTRIPZ | Hela Kyoto |
| cDD128 | K16/R Hbp1 Δ1-10 | pTRIPZ | Hela Kyoto |
| cDD90 | WDR26 resistant | pTRIPZ | Hela Kyoto |
| cDD114 | HSS-MAEA | pTRIPZ | Hela Kyoto |
| cDD129 | Twa1-HSS | pTRIPZ | Hela Kyoto |
|  |  |  |  |
|  |  |  |  |
| Lentivirus NAME | Insert | Backbone | Gene |
| vDD100 | pDD100 | lentiCRISPR v2 | WDR26 |
| vDD101 | pDD101 | lentiCRISPR v2 | WDR26 |
| vDD102 | pDD102 | lentiCRISPR v2 | WDR26 |
| vDD105 | pDD105 | lentiCRISPR v2 | Control |
| vDD121 | pDD121 | lentiCRISPR v2 | MAEA |
| vDD122 | pDD122 | lentiCRISPR v2 | MAEA |
| vDD123 | pDD123 | lentiCRISPR v2 | MAEA |
| vDD124 | pDD124 | lentiCRISPR v2 | MAEA |
| vDD125 | pDD125 | lentiCRISPR v2 | MAEA |
| vDD116 | pDD116 | lentiCRISPR v2 | Luciferase Control |
| vDD1 | pDD90 | pTRIPZ | WDR26 resistant |
| vDD2 | pDD149 | pTRIPZ | Hbp1 canonical |
| vDD7 | pDD128 | pTRIPZ | K16/R Hbp1 Δ1-10 |
| vDD8 | pDD127 | pTRIPZ | P11/G Hbp1 Δ1-10 |
| pDD10 | pDD126 | pTRIPZ | Hbp1 Δ1-10 |
| vDD30 | pDD114 | pTRIPZ | HSS-MAEA |
| vDD33 | pDD129 | pTRIPZ | Twa1-HSS |
